# Search for massive long-lived particles decaying semileptonically in the LHCb detector

**DOI:** 10.1140/epjc/s10052-017-4744-6

**Published:** 2017-04-10

**Authors:** R. Aaij, B. Adeva, M. Adinolfi, Z. Ajaltouni, S. Akar, J. Albrecht, F. Alessio, M. Alexander, S. Ali, G. Alkhazov, P. Alvarez Cartelle, A. A. Alves, S. Amato, S. Amerio, Y. Amhis, L. An, L. Anderlini, G. Andreassi, M. Andreotti, J. E. Andrews, R. B. Appleby, F. Archilli, P. d’Argent, J. Arnau Romeu, A. Artamonov, M. Artuso, E. Aslanides, G. Auriemma, M. Baalouch, I. Babuschkin, S. Bachmann, J. J. Back, A. Badalov, C. Baesso, S. Baker, W. Baldini, R. J. Barlow, C. Barschel, S. Barsuk, W. Barter, M. Baszczyk, V. Batozskaya, B. Batsukh, V. Battista, A. Bay, L. Beaucourt, J. Beddow, F. Bedeschi, I. Bediaga, L. J. Bel, V. Bellee, N. Belloli, K. Belous, I. Belyaev, E. Ben-Haim, G. Bencivenni, S. Benson, J. Benton, A. Berezhnoy, R. Bernet, A. Bertolin, C. Betancourt, F. Betti, M.-O. Bettler, M. van Beuzekom, Ia. Bezshyiko, S. Bifani, P. Billoir, T. Bird, A. Birnkraut, A. Bitadze, A. Bizzeti, T. Blake, F. Blanc, J. Blouw, S. Blusk, V. Bocci, T. Boettcher, A. Bondar, N. Bondar, W. Bonivento, I. Bordyuzhin, A. Borgheresi, S. Borghi, M. Borisyak, M. Borsato, F. Bossu, M. Boubdir, T. J. V. Bowcock, E. Bowen, C. Bozzi, S. Braun, M. Britsch, T. Britton, J. Brodzicka, E. Buchanan, C. Burr, A. Bursche, J. Buytaert, S. Cadeddu, R. Calabrese, M. Calvi, M. Calvo Gomez, A. Camboni, P. Campana, D. H. Campora Perez, L. Capriotti, A. Carbone, G. Carboni, R. Cardinale, A. Cardini, P. Carniti, L. Carson, K. Carvalho Akiba, G. Casse, L. Cassina, L. Castillo Garcia, M. Cattaneo, Ch. Cauet, G. Cavallero, R. Cenci, D. Chamont, M. Charles, Ph. Charpentier, G. Chatzikonstantinidis, M. Chefdeville, S. Chen, S.-F. Cheung, V. Chobanova, M. Chrzaszcz, X. Cid Vidal, G. Ciezarek, P. E. L. Clarke, M. Clemencic, H. V. Cliff, J. Closier, V. Coco, J. Cogan, E. Cogneras, V. Cogoni, L. Cojocariu, G. Collazuol, P. Collins, A. Comerma-Montells, A. Contu, A. Cook, G. Coombs, S. Coquereau, G. Corti, M. Corvo, C. M. Costa Sobral, B. Couturier, G. A. Cowan, D. C. Craik, A. Crocombe, M. Cruz Torres, S. Cunliffe, R. Currie, C. D’Ambrosio, F. Da Cunha Marinho, E. Dall’Occo, J. Dalseno, P. N. Y. David, A. Davis, O. De Aguiar Francisco, K. De Bruyn, S. De Capua, M. De Cian, J. M. De Miranda, L. De Paula, M. De Serio, P. De Simone, C.-T. Dean, D. Decamp, M. Deckenhoff, L. Del Buono, M. Demmer, A. Dendek, D. Derkach, O. Deschamps, F. Dettori, B. Dey, A. Di Canto, H. Dijkstra, F. Dordei, M. Dorigo, A. Dosil Suárez, A. Dovbnya, K. Dreimanis, L. Dufour, G. Dujany, K. Dungs, P. Durante, R. Dzhelyadin, A. Dziurda, A. Dzyuba, N. Déléage, S. Easo, M. Ebert, U. Egede, V. Egorychev, S. Eidelman, S. Eisenhardt, U. Eitschberger, R. Ekelhof, L. Eklund, S. Ely, S. Esen, H. M. Evans, T. Evans, A. Falabella, N. Farley, S. Farry, R. Fay, D. Fazzini, D. Ferguson, A. Fernandez Prieto, F. Ferrari, F. Ferreira Rodrigues, M. Ferro-Luzzi, S. Filippov, R. A. Fini, M. Fiore, M. Fiorini, M. Firlej, C. Fitzpatrick, T. Fiutowski, F. Fleuret, K. Fohl, M. Fontana, F. Fontanelli, D. C. Forshaw, R. Forty, V. Franco Lima, M. Frank, C. Frei, J. Fu, W. Funk, E. Furfaro, C. Färber, A. Gallas Torreira, D. Galli, S. Gallorini, S. Gambetta, M. Gandelman, P. Gandini, Y. Gao, L. M. Garcia Martin, J. García Pardiñas, J. Garra Tico, L. Garrido, P. J. Garsed, D. Gascon, C. Gaspar, L. Gavardi, G. Gazzoni, D. Gerick, E. Gersabeck, M. Gersabeck, T. Gershon, Ph. Ghez, S. Gianì, V. Gibson, O. G. Girard, L. Giubega, K. Gizdov, V. V. Gligorov, D. Golubkov, A. Golutvin, A. Gomes, I. V. Gorelov, C. Gotti, M. Grabalosa Gándara, R. Graciani Diaz, L. A. Granado Cardoso, E. Graugés, E. Graverini, G. Graziani, A. Grecu, P. Griffith, L. Grillo, B. R. Gruberg Cazon, O. Grünberg, E. Gushchin, Yu. Guz, T. Gys, C. Göbel, T. Hadavizadeh, C. Hadjivasiliou, G. Haefeli, C. Haen, S. C. Haines, S. Hall, B. Hamilton, X. Han, S. Hansmann-Menzemer, N. Harnew, S. T. Harnew, J. Harrison, M. Hatch, J. He, T. Head, A. Heister, K. Hennessy, P. Henrard, L. Henry, E. van Herwijnen, M. Heß, A. Hicheur, D. Hill, C. Hombach, H. Hopchev, W. Hulsbergen, T. Humair, M. Hushchyn, N. Hussain, D. Hutchcroft, M. Idzik, P. Ilten, R. Jacobsson, A. Jaeger, J. Jalocha, E. Jans, A. Jawahery, F. Jiang, M. John, D. Johnson, C. R. Jones, C. Joram, B. Jost, N. Jurik, S. Kandybei, W. Kanso, M. Karacson, J. M. Kariuki, S. Karodia, M. Kecke, M. Kelsey, M. Kenzie, T. Ketel, E. Khairullin, B. Khanji, C. Khurewathanakul, T. Kirn, S. Klaver, K. Klimaszewski, S. Koliiev, M. Kolpin, I. Komarov, R. F. Koopman, P. Koppenburg, A. Kosmyntseva, A. Kozachuk, M. Kozeiha, L. Kravchuk, K. Kreplin, M. Kreps, P. Krokovny, F. Kruse, W. Krzemien, W. Kucewicz, M. Kucharczyk, V. Kudryavtsev, A. K. Kuonen, K. Kurek, T. Kvaratskheliya, D. Lacarrere, G. Lafferty, A. Lai, G. Lanfranchi, C. Langenbruch, T. Latham, C. Lazzeroni, R. Le Gac, J. van Leerdam, A. Leflat, J. Lefrançois, R. Lefèvre, F. Lemaitre, E. Lemos Cid, O. Leroy, T. Lesiak, B. Leverington, T. Li, Y. Li, T. Likhomanenko, R. Lindner, C. Linn, F. Lionetto, X. Liu, D. Loh, I. Longstaff, J. H. Lopes, D. Lucchesi, M. Lucio Martinez, H. Luo, A. Lupato, E. Luppi, O. Lupton, A. Lusiani, X. Lyu, F. Machefert, F. Maciuc, O. Maev, K. Maguire, S. Malde, A. Malinin, T. Maltsev, G. Manca, G. Mancinelli, P. Manning, J. Maratas, J. F. Marchand, U. Marconi, C. Marin Benito, P. Marino, J. Marks, G. Martellotti, M. Martin, M. Martinelli, D. Martinez Santos, F. Martinez Vidal, D. Martins Tostes, L. M. Massacrier, A. Massafferri, R. Matev, A. Mathad, Z. Mathe, C. Matteuzzi, A. Mauri, B. Maurin, A. Mazurov, M. McCann, J. McCarthy, A. McNab, R. McNulty, B. Meadows, F. Meier, M. Meissner, D. Melnychuk, M. Merk, A. Merli, E. Michielin, D. A. Milanes, M.-N. Minard, D. S. Mitzel, A. Mogini, J. Molina Rodriguez, I. A. Monroy, S. Monteil, M. Morandin, P. Morawski, A. Mordà, M. J. Morello, J. Moron, A. B. Morris, R. Mountain, F. Muheim, M. Mulder, M. Mussini, B. Muster, D. Müller, J. Müller, K. Müller, V. Müller, P. Naik, T. Nakada, R. Nandakumar, A. Nandi, I. Nasteva, M. Needham, N. Neri, S. Neubert, N. Neufeld, M. Neuner, T. D. Nguyen, C. Nguyen-Mau, S. Nieswand, R. Niet, N. Nikitin, T. Nikodem, A. Novoselov, D. P. O’Hanlon, A. Oblakowska-Mucha, V. Obraztsov, S. Ogilvy, R. Oldeman, C. J. G. Onderwater, J. M. Otalora Goicochea, A. Otto, P. Owen, A. Oyanguren, P. R. Pais, A. Palano, F. Palombo, M. Palutan, J. Panman, A. Papanestis, M. Pappagallo, L. L. Pappalardo, W. Parker, C. Parkes, G. Passaleva, A. Pastore, G. D. Patel, M. Patel, C. Patrignani, A. Pearce, A. Pellegrino, G. Penso, M. Pepe Altarelli, S. Perazzini, P. Perret, L. Pescatore, K. Petridis, A. Petrolini, A. Petrov, M. Petruzzo, E. Picatoste Olloqui, B. Pietrzyk, M. Pikies, D. Pinci, A. Pistone, A. Piucci, S. Playfer, M. Plo Casasus, T. Poikela, F. Polci, A. Poluektov, I. Polyakov, E. Polycarpo, G. J. Pomery, A. Popov, D. Popov, B. Popovici, S. Poslavskii, C. Potterat, E. Price, J. D. Price, J. Prisciandaro, A. Pritchard, C. Prouve, V. Pugatch, A. Puig Navarro, G. Punzi, W. Qian, R. Quagliani, B. Rachwal, J. H. Rademacker, M. Rama, M. Ramos Pernas, M. S. Rangel, I. Raniuk, F. Ratnikov, G. Raven, F. Redi, S. Reichert, A. C. dos Reis, C. Remon Alepuz, V. Renaudin, S. Ricciardi, S. Richards, M. Rihl, K. Rinnert, V. Rives Molina, P. Robbe, A. B. Rodrigues, E. Rodrigues, J. A. Rodriguez Lopez, P. Rodriguez Perez, A. Rogozhnikov, S. Roiser, A. Rollings, V. Romanovskiy, A. Romero Vidal, J. W. Ronayne, M. Rotondo, M. S. Rudolph, T. Ruf, P. Ruiz Valls, J. J. Saborido Silva, E. Sadykhov, N. Sagidova, B. Saitta, V. Salustino Guimaraes, C. Sanchez Mayordomo, B. Sanmartin Sedes, R. Santacesaria, C. Santamarina Rios, M. Santimaria, E. Santovetti, A. Sarti, C. Satriano, A. Satta, D. M. Saunders, D. Savrina, S. Schael, M. Schellenberg, M. Schiller, H. Schindler, M. Schlupp, M. Schmelling, T. Schmelzer, B. Schmidt, O. Schneider, A. Schopper, K. Schubert, M. Schubiger, M.-H. Schune, R. Schwemmer, B. Sciascia, A. Sciubba, A. Semennikov, A. Sergi, N. Serra, J. Serrano, L. Sestini, P. Seyfert, M. Shapkin, I. Shapoval, Y. Shcheglov, T. Shears, L. Shekhtman, V. Shevchenko, B. G. Siddi, R. Silva Coutinho, L. Silva de Oliveira, G. Simi, S. Simone, M. Sirendi, N. Skidmore, T. Skwarnicki, E. Smith, I. T. Smith, J. Smith, M. Smith, H. Snoek, l. Soares Lavra, M. D. Sokoloff, F. J. P. Soler, B. Souza De Paula, B. Spaan, P. Spradlin, S. Sridharan, F. Stagni, M. Stahl, S. Stahl, P. Stefko, S. Stefkova, O. Steinkamp, S. Stemmle, O. Stenyakin, S. Stevenson, S. Stoica, S. Stone, B. Storaci, S. Stracka, M. Straticiuc, U. Straumann, L. Sun, W. Sutcliffe, K. Swientek, V. Syropoulos, M. Szczekowski, T. Szumlak, S. T’Jampens, A. Tayduganov, T. Tekampe, M. Teklishyn, G. Tellarini, F. Teubert, E. Thomas, J. van Tilburg, M. J. Tilley, V. Tisserand, M. Tobin, S. Tolk, L. Tomassetti, D. Tonelli, S. Topp-Joergensen, F. Toriello, E. Tournefier, S. Tourneur, K. Trabelsi, M. Traill, M. T. Tran, M. Tresch, A. Trisovic, A. Tsaregorodtsev, P. Tsopelas, A. Tully, N. Tuning, A. Ukleja, A. Ustyuzhanin, U. Uwer, C. Vacca, V. Vagnoni, A. Valassi, S. Valat, G. Valenti, A. Vallier, R. Vazquez Gomez, P. Vazquez Regueiro, S. Vecchi, M. van Veghel, J. J. Velthuis, M. Veltri, G. Veneziano, A. Venkateswaran, M. Vernet, M. Vesterinen, B. Viaud, D. Vieira, M. Vieites Diaz, H. Viemann, X. Vilasis-Cardona, M. Vitti, V. Volkov, A. Vollhardt, B. Voneki, A. Vorobyev, V. Vorobyev, C. Voß, J. A. de Vries, C. Vázquez Sierra, R. Waldi, C. Wallace, R. Wallace, J. Walsh, J. Wang, D. R. Ward, H. M. Wark, N. K. Watson, D. Websdale, A. Weiden, M. Whitehead, J. Wicht, G. Wilkinson, M. Wilkinson, M. Williams, M. P. Williams, M. Williams, T. Williams, F. F. Wilson, J. Wimberley, J. Wishahi, W. Wislicki, M. Witek, G. Wormser, S. A. Wotton, K. Wraight, K. Wyllie, Y. Xie, Z. Xing, Z. Xu, Z. Yang, Y. Yao, H. Yin, J. Yu, X. Yuan, O. Yushchenko, K. A. Zarebski, M. Zavertyaev, L. Zhang, Y. Zhang, Y. Zhang, A. Zhelezov, Y. Zheng, X. Zhu, V. Zhukov, S. Zucchelli, LHCb Collaboration

**Affiliations:** 1 Centro Brasileiro de Pesquisas Físicas (CBPF), Rio de Janeiro, Brazil; 2 Universidade Federal do Rio de Janeiro (UFRJ), Rio de Janeiro, Brazil; 3 Center for High Energy Physics, Tsinghua University, Beijing, China; 4 LAPP, Université Savoie Mont-Blanc, CNRS/IN2P3, Annecy-le-Vieux, France; 5 Clermont Université, Université Blaise Pascal, CNRS/IN2P3, LPC, Clermont-Ferrand, France; 6 CPPM, Aix-Marseille Université, CNRS/IN2P3, Marseille, France; 7 LAL, Université Paris-Sud, CNRS/IN2P3, Orsay, France; 8 LPNHE, Université Pierre et Marie Curie, Université Paris Diderot, CNRS/IN2P3, Paris, France; 9 I. Physikalisches Institut, RWTH Aachen University, Aachen, Germany; 10 Fakultät Physik, Technische Universität Dortmund, Dortmund, Germany; 11 Max-Planck-Institut für Kernphysik (MPIK), Heidelberg, Germany; 12 Physikalisches Institut, Ruprecht-Karls-Universität Heidelberg, Heidelberg, Germany; 13 School of Physics, University College Dublin, Dublin, Ireland; 14 Sezione INFN di Bari, Bari, Italy; 15 Sezione INFN di Bologna, Bologna, Italy; 16 Sezione INFN di Cagliari, Cagliari, Italy; 17 Sezione INFN di Ferrara, Ferrara, Italy; 18 Sezione INFN di Firenze, Florence, Italy; 19 Laboratori Nazionali dell’INFN di Frascati, Frascati, Italy; 20 Sezione INFN di Genova, Genoa, Italy; 21 Sezione INFN di Milano Bicocca, Milan, Italy; 22 Sezione INFN di Milano, Milan, Italy; 23 Sezione INFN di Padova, Padua, Italy; 24 Sezione INFN di Pisa, Pisa, Italy; 25 Sezione INFN di Roma Tor Vergata, Rome, Italy; 26 Sezione INFN di Roma La Sapienza, Rome, Italy; 27 Henryk Niewodniczanski Institute of Nuclear Physics Polish Academy of Sciences, Kraków, Poland; 28 Faculty of Physics and Applied Computer Science, AGH-University of Science and Technology, Kraków, Poland; 29 National Center for Nuclear Research (NCBJ), Warsaw, Poland; 30 Horia Hulubei National Institute of Physics and Nuclear Engineering, Bucharest-Magurele, Romania; 31 Petersburg Nuclear Physics Institute (PNPI), Gatchina, Russia; 32 Institute of Theoretical and Experimental Physics (ITEP), Moscow, Russia; 33 Institute of Nuclear Physics, Moscow State University (SINP MSU), Moscow, Russia; 34 Institute for Nuclear Research of the Russian Academy of Sciences (INR RAN), Moscow, Russia; 35 Yandex School of Data Analysis, Moscow, Russia; 36 Budker Institute of Nuclear Physics (SB RAS), Novosibirsk, Russia; 37 Institute for High Energy Physics (IHEP), Protvino, Russia; 38 ICCUB, Universitat de Barcelona, Barcelona, Spain; 39 Universidad de Santiago de Compostela, Santiago de Compostela, Spain; 40 European Organization for Nuclear Research (CERN), Geneva, Switzerland; 41 Institute of Physics, Ecole Polytechnique Fédérale de Lausanne (EPFL), Lausanne, Switzerland; 42 Physik-Institut, Universität Zürich, Zurich, Switzerland; 43 Nikhef National Institute for Subatomic Physics, Amsterdam, The Netherlands; 44 Nikhef National Institute for Subatomic Physics, VU University Amsterdam, Amsterdam, The Netherlands; 45 NSC Kharkiv Institute of Physics and Technology (NSC KIPT), Kharkiv, Ukraine; 46 Institute for Nuclear Research of the National Academy of Sciences (KINR), Kiev, Ukraine; 47 University of Birmingham, Birmingham, UK; 48 H.H. Wills Physics Laboratory, University of Bristol, Bristol, UK; 49 Cavendish Laboratory, University of Cambridge, Cambridge, UK; 50 Department of Physics, University of Warwick, Coventry, UK; 51 STFC Rutherford Appleton Laboratory, Didcot, UK; 52 School of Physics and Astronomy, University of Edinburgh, Edinburgh, UK; 53 School of Physics and Astronomy, University of Glasgow, Glasgow, UK; 54 Oliver Lodge Laboratory, University of Liverpool, Liverpool, UK; 55 Imperial College London, London, UK; 56 School of Physics and Astronomy, University of Manchester, Manchester, UK; 57 Department of Physics, University of Oxford, Oxford, UK; 58 Massachusetts Institute of Technology, Cambridge, MA USA; 59 University of Cincinnati, Cincinnati, OH USA; 60 University of Maryland, College Park, MD USA; 61 Syracuse University, Syracuse, NY USA; 62 Pontifícia Universidade Católica do Rio de Janeiro (PUC-Rio), Rio de Janeiro, Brazil; 63 University of Chinese Academy of Sciences, Beijing, China; 64 School of Physics and Technology, Wuhan University, Wuhan, China; 65 Institute of Particle Physics, Central China Normal University, Wuhan, Hubei China; 66 Departamento de Fisica, Universidad Nacional de Colombia, Bogotá, Colombia; 67 Institut für Physik, Universität Rostock, Rostock, Germany; 68 National Research Centre Kurchatov Institute, Moscow, Russia; 69 Instituto de Fisica Corpuscular (IFIC), Universitat de Valencia-CSIC, Valencia, Spain; 70 Van Swinderen Institute, University of Groningen, Groningen, The Netherlands; 71 CERN, 1211 Geneva 23, Switzerland

## Abstract

A search is presented for massive long-lived particles decaying into a muon and two quarks. The dataset consists of proton-proton interactions at centre-of-mass energies of 7 and 8 TeV, corresponding to integrated luminosities of 1 and 2$\,\hbox {fb}^{-1}$, respectively. The analysis is performed assuming a set of production mechanisms with simple topologies, including the production of a Higgs-like particle decaying into two long-lived particles. The mass range from 20 to 80 $\mathrm{{GeV}}/c^2$ and lifetimes from 5 to 100${\,\mathrm{{ps}}}$ are explored. Results are also interpreted in terms of neutralino production in different R-Parity violating supersymmetric models, with masses in the 23–198 GeV/$c^2$ range. No excess above the background expectation is observed and upper limits are set on the production cross-section for various points in the parameter space of theoretical models.

## Introduction

Supersymmetry (SUSY) is one of the most popular extensions of the Standard Model, which solves the hierarchy problem, can unify the gauge couplings and could provide dark matter candidates. The minimal supersymmetric standard model (MSSM) is the simplest phenomenologically viable realization of SUSY [1, 2]. The present study focuses on a subset of models featuring massive long-lived particles (LLP) with a measurable flight distance [3, 4]. LLP searches have been performed by Tevatron and LHC experiments [5–12], often using the Hidden Valley framework [4] as a benchmark model (see also the study of Ref. [13]). The LHCb detector probes the forward rapidity region which is only partially covered by the other LHC experiments, and triggers on particles with low transverse momenta, which allows the experiment to explore relatively small LLP masses.

In this paper a search for massive long-lived particles is presented, using proton-proton collision data collected by the LHCb detector at $\sqrt{s} =7$ and 8 TeV, corresponding to integrated luminosities of 1 and 2 $\,\hbox {fb}^{-1}$, respectively. The event topology considered in this study is a displaced vertex with several tracks including a high $p_{\mathrm { T}}$ muon. This topology is found in the context of the minimal super-gravity (mSUGRA) realisation of the MSSM, with R-parity violation [14], in which the neutralino can decay into a muon and two jets. Neutralinos can be produced by a variety of processes. In this paper four simple production mechanisms with representative topologies and kinematics are considered, with the assumed LLP mass in the range 20–80 $\mathrm{{GeV}}/c^2$. The LLP lifetime range considered is 5–100${\,\mathrm{{ps}}}$, i.e. larger than the typical *b*-hadron lifetime. It corresponds to an average flight distance of up to 30$\mathrm { \,cm}$, well inside the LHCb vertex detector. One of the production mechanisms considered in detail is the decay into two LLPs of a Higgs-like particle with an assumed mass between 50 and 130 $\mathrm{{GeV}}/c^2$, i.e. in a range which includes the mass of the scalar boson discovered by the ATLAS and CMS experiments [15, 16]. In addition, inclusive analyses are performed assuming the full set of neutralino production mechanisms available in Pythia  6 [17]. In this case the LLP mass explored is in the range 23–198 $\mathrm{{GeV}}/c^2$, inspired by Ref. [13], and different combinations of gluino and squark masses are studied.

## Detector description

The LHCb detector [18, 19] is a single-arm forward spectrometer covering the pseudorapidity range $$2<\eta <5$$, designed for the study of particles containing $$b $$ or $$\mathrm {c} $$ quarks. The detector includes a high-precision tracking system consisting of a silicon-strip vertex detector surrounding the *pp* interaction region (VELO), a large-area silicon-strip detector located upstream of a dipole magnet with a bending power of about $$4{\mathrm {\,Tm}}$$, and three stations of silicon-strip detectors and straw drift tubes, placed downstream of the magnet. The tracking system provides a measurement of momentum, $${p}$$, of charged particles with a relative uncertainty that varies from 0.5% at low momentum to 1.0% at 200$${\mathrm {\,GeV}/c}$$. The minimum distance of a track to a primary vertex (PV), the impact parameter $$d_\mathrm{{IP}}$$, is measured with a resolution of $$(15+29/p_{\mathrm { T}}){\,\upmu \mathrm {m}} $$, where $$p_{\mathrm { T}}$$ is the component of the momentum transverse to the beam axis, in $${\mathrm {\,GeV}/c}$$. Different types of charged hadrons are distinguished using information from two ring-imaging Cherenkov detectors. Photons, electrons and hadrons are identified by a calorimeter system consisting of scintillating-pad and preshower detectors, an electromagnetic calorimeter and a hadronic calorimeter. Muons are identified by a system composed of alternating layers of iron and multiwire proportional chambers. The online event selection is performed by a trigger [20], which consists of a hardware stage based on information from the calorimeter and muon systems, followed by a software stage which runs a simplified version of the offline event reconstruction.

## Event generation and detector simulation

Several sets of simulated events are used to design and optimize the signal selection and to estimate the detection efficiency. Proton-proton collisions are generated in Pythia  6 with a specific LHCb configuration [21], and with parton density functions taken from CTEQ6L [22]. The LLP signal in this framework is represented by the lightest neutralino $\tilde{\chi }^{0}_{1}$, with mass $m_\mathrm{{LLP}}$ and lifetime $\tau _\mathrm{{LLP}}$. It is allowed to decay into two quarks and a muon. Decays to all quark pairs are assumed to have identical branching fractions except for those involving a top quark, which are neglected.

Two separate detector simulations are used to produce signal models: a full simulation, where the interaction of the generated particles with the detector is based on Geant4 [23–25], and a fast simulation. In Geant4, the detector and its response are implemented as described in Ref. [26]. In the fast simulation, which is used to cover a broader parameter space of the theoretical models, the charged particles falling into the geometrical acceptance of the detector are processed by the vertex reconstruction algorithm. The simulation accounts for the effects of the material veto described in the next section. The program also provides parameterised particle momenta resolutions, but it is found that these resolutions have no significant impact on the LLP mass reconstruction, nor on the signal detection efficiency. The fast simulation is validated by comparison with the full simulation. The distributions for mass, momentum and transverse momentum of the reconstructed LLP and for the reconstructed decay vertex position are in excellent agreement, as well as the muon momentum and its impact parameter to the PV. The detection efficiencies predicted by the full and the fast simulation differ by less than 5%.

**Fig. 1:**
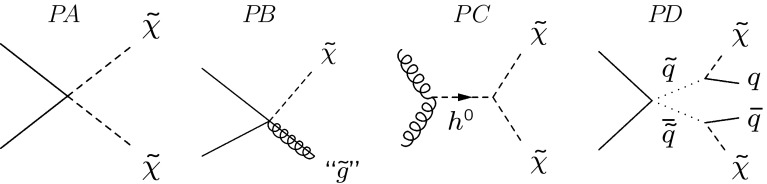
Four topologies considered as representative LLP production mechanisms: $P\!A$ non-resonant direct double LLP production, $P\!B$ single LLP production, $P\!\,C$ double LLP production from the decay of a Higgs-like boson, $P\!D$ double LLP indirect production via squarks

Two LLP production scenarios are considered. In the first, the signal samples are generated assuming the full set of neutralino production processes available in Pythia. In particular, nine models are fully simulated with the parameters given in the Appendix, Table 4. Other points in the parameter space of the theoretical models are studied with the fast simulation, covering the $m_\mathrm{{LLP}}$ range 23–198 $\mathrm{{GeV}}/c^2$. These models are referred to as “LV” (for lepton number violation) followed by the LLP mass in  $\mathrm{{GeV}}/c^2$ and lifetime (e.g. LV98 10${\,\mathrm{{ps}}}$). For the second scenario, the four production mechanisms depicted in Fig. 1, labelled $P\!A$, $P\!B$, $P\!\,C$, and $P\!D$, are selected and studied independently with the fast simulation. The LLP, represented by the neutralino, subsequently decays into two quarks and a muon. The processes $P\!A$, $P\!\,C$, and $P\!D$ have two LLPs in the final state. In processes $P\!\,C$ and $P\!D$ two LLPs are produced by the decay of a Higgs-like particle of mass $m_\mathrm{h^0}$, and by the decay of squarks of mass $m_{\tilde{\mathrm{q}}}$, respectively. In process $P\!B$ a single LLP is produced recoiling against an object labelled as a “gluino”, of mass $m_{\text {``}\tilde{\mathrm{g}}\text {''}}$. In order to control the kinematic conditions, the particles generated in these processes are constrained to be on-shell and the “gluino” of option $P\!B$ is stable. Since LHCb is most sensitive to relatively low LLP masses, only $m_\mathrm{{LLP}}$ values below 80 $\mathrm{{GeV}}/c^2$ are considered.

The background from direct production of heavy quarks, as well as from ${\mathrm {W}}$ and ${\mathrm {Z}}$ boson decays, is studied using the full simulation. A sample of $9 \times 10^6$ inclusive ${\mathrm {c}} {\overline{{\mathrm {c}}}}$ events with at least two $\mathrm {c}$ hadrons in $1.5<\eta <5.0$, and another sample of about $5 \times 10^5$ ${\mathrm {t}} {\overline{{\mathrm {t}}}}$ events with at least one muon in $1.5<\eta <5.0$ and $p_{\mathrm { T}} >10{\mathrm {\,GeV}/c}$ were produced. Several million simulated events are available with production of ${\mathrm {W}}$ and ${\mathrm {Z}}$ bosons. The most relevant background in this analysis is from ${b} {\overline{{b}}}$ events. The available simulated inclusive ${b} {\overline{{b}}}$ events are not numerous enough to cover the high-$p_{\mathrm { T}}$ muon kinematic region required in this analysis. To enhance the ${b} {\overline{{b}}}$ background statistics, a dedicated sample of $2.14 \times 10^5$ simulated events has been produced with a minimum parton $\hat{p}_\mathrm{T}$ of 20${\mathrm {\,GeV}/c}$ and requiring a muon with $p_{\mathrm { T}} >12{\mathrm {\,GeV}/c}$ in $1.5<\eta <5.0$. As a consequence of limitations in the available computing power, only ${b} {\overline{{b}}}$ events with $\sqrt{s} =7\,\mathrm{{TeV}}$ have been fully simulated. Despite the considerable increase of generation efficiency, all the simulated ${b} {\overline{{b}}}$ events are rejected by the multivariate analysis presented in the next section. Therefore a data-driven approach is employed for the final background estimation.

## Event selection

Signal events are selected by requiring a displaced high-multiplicity vertex with one associated isolated high-$p_{\mathrm { T}}$ muon, since, due to the larger particle mass, muons from LLP decays are expected to have larger transverse momenta and to be more isolated than muons from hadron decays.

The events from *pp* collisions are selected online by a trigger requiring muons with $p_{\mathrm { T}} >10{\mathrm {\,GeV}/c}$. Primary vertices and displaced vertices are reconstructed offline from charged particle tracks [27] with a minimum reconstructed $p_{\mathrm { T}}$ of 100${\mathrm {\,MeV}/c}$. Genuine PVs are identified by a small radial distance from the beam axis, $R_\mathrm{{xy}} <0.3$ mm. The offline analysis requires that the triggering muon has an impact parameter to all PVs of $d_\mathrm{{IP}} >0.25\,{\mathrm {mm}}$ and $p_{\mathrm { T}} >12$ ${\mathrm {\,GeV}/c}$. To suppress the background due to kaons or pions punching through the calorimeters and being misidentified as muons, the corresponding energy deposit in the calorimeters must be less than 4% of the muon energy. To preserve enough background events in the signal-free region for the signal determination algorithm described in Sect. 5, no isolation requirement is applied at this stage. Secondary vertices are selected by requiring $R_\mathrm{{xy}} >0.55$ mm, at least four tracks in the forward direction (i.e. in the direction of the spectrometer) including the muon and no tracks in the backward direction. The total invariant mass of the tracks coming from a selected vertex must be larger than 4.5 $\mathrm{{GeV}}/c^2$. Particles interacting with the detector material are an important source of background. A geometric veto is used to reject events with vertices in regions occupied by detector material [28].

**Fig. 2:**
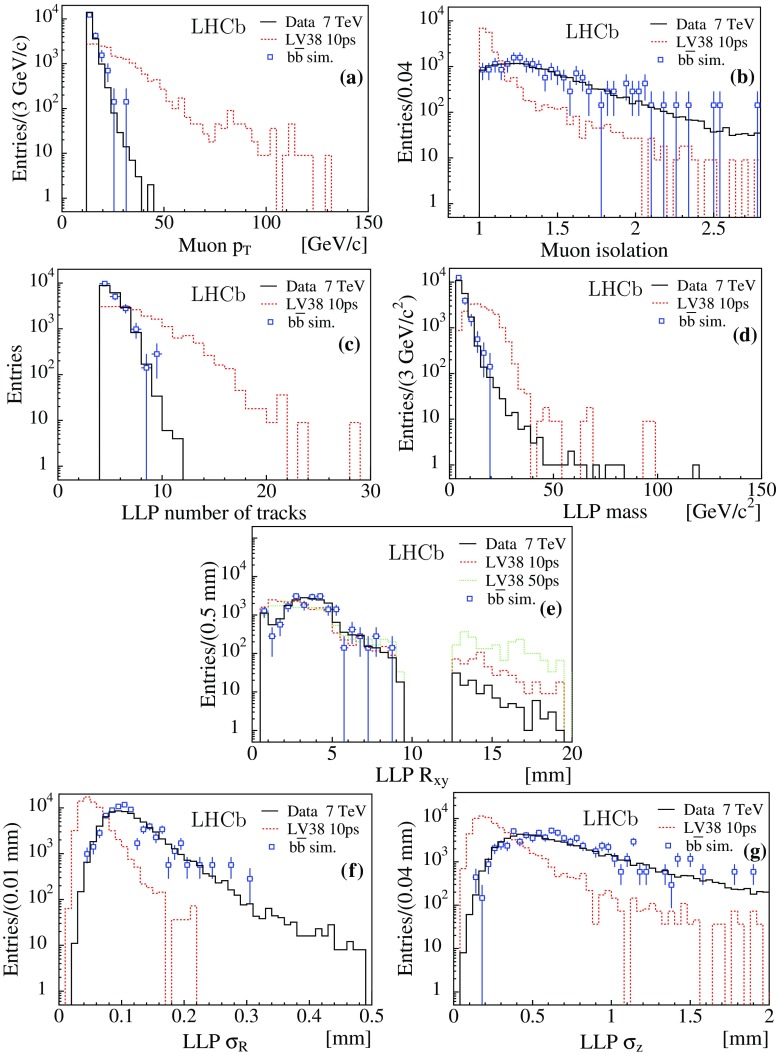
Distributions for the 7 TeV dataset (*black histogram*) compared to simulated ${b} {\overline{{b}}}$ events (*blue squares* with *error bars*), showing **a** transverse momentum and **b** isolation of the muon, **c** number of tracks of the displaced vertex, **d** reconstructed mass, **e** radial position of the vertex, **f**, **g** vertex fit uncertainties in the radial and z direction. The fully simulated signal distributions for LV38 10 ps are shown (*red dashed histograms*), as well as LV38 50 ps (*green dotted histogram*) in (**e**). The distributions from simulation are normalised to the number of data entries

The number of data events selected is 18 925 (53 331) in the $$7\,\mathrm{{TeV}}$$ ($$\mathrm 8\,\mathrm{{TeV}}$$) datasets. Less than 1% of the events have more than one candidate vertex, in which case the candidate with the highest-$$p_{\mathrm { T}}$$ muon is chosen. According to the simulation, the background is largely dominated by $${b} {\overline{{b}}} $$ events, while the contribution from the decays of $${\mathrm {W}} $$ and $${\mathrm {Z}} $$ bosons is of the order of 10 events. All simulated $${\mathrm {c}} {\overline{{\mathrm {c}}}} $$ and $${\mathrm {t}} {\overline{{\mathrm {t}}}} $$ events are rejected. The $${b} {\overline{{b}}} $$ cross-section value measured by LHCb, $$ 288 \pm 4 \pm 48$$ $$\mu $$b [29–32], predicts $$(15 \pm 3)\times 10^3$$ events for the $$7\,\mathrm{{TeV}}$$ dataset, after selection. The value for the $$\mathrm 8\,\mathrm{{TeV}}$$ dataset is $$(52 \pm 10)\times 10^3$$. The extrapolation of the cross-section from 7 to $$\mathrm 8\,\mathrm{{TeV}}$$ is obtained from POWHEG [33–35], while Pythia is used to obtain the detection efficiency. The candidate yields for the two datasets are consistent with a dominant $${b} {\overline{{b}}} $$ composition of the background. This is confirmed by the study of the shapes of the distributions of the relevant observables. Figure 2 compares the distributions for the $$7\,\mathrm{{TeV}}$$ dataset and for the 135 simulated $${b} {\overline{{b}}} $$ events surviving the selection. For illustration, the shapes of simulated LV38 10 ps signal events are superimposed on all the distributions, as well as the expected shape for LV38 50 ps on the $$R_{\rm xy}$$ distribution. The muon isolation variable is defined as the sum of the energy of tracks surrounding the muon direction, including the muon itself, in a cone of radius $$R_{\eta \phi }= 0.3$$ in the pseudorapidity-azimuthal angle $$(\eta , \phi )$$ space, divided by the energy of the muon track. The corresponding distribution is shown in Fig. 2b. A muon isolation value of unity denotes a fully isolated muon. As expected, the muon from the signal is found to be more isolated than the hadronic background. Figure 2e presents the radial distribution of the displaced vertices; the drop in the number of candidates with a vertex above $$R_\mathrm{{xy}} \sim 5\,{\mathrm {mm}} $$ is due to the material veto. From simulation, the veto introduces a loss of efficiency of 13% (42%) for the detection of LLPs with a $$30\,\mathrm{{GeV}}/c^2 $$ mass and a 10$${\,\mathrm{{ps}}}$$ (100$${\,\mathrm{{ps}}}$$) lifetime. The radial ($$\sigma _\mathrm{R}$$) and longitudinal ($$\sigma _\mathrm{z}$$, parallel to the beam) uncertainties provided by the LLP vertex fit are shown in Fig. 2f, g. Larger uncertainties are expected from the vertex fits of candidates from $${b} {\overline{{b}}} $$ events compared to signal LLPs. The former are more boosted and produce more narrowly collimated tracks, while the relatively heavier signal LLPs decay into more divergent tracks. This effect decreases when $$m_\mathrm{{LLP}}$$ approaches the mass of $$b $$-quark hadrons.

A multivariate analysis based on a multi-layer perceptron (MLP) [36, 37] is used to further purify the data sample. The MLP input variables are the muon $p_{\mathrm { T}}$ and impact parameter, the number of charged particle tracks used to reconstruct the LLP, the vertex radial distance $R_\mathrm{{xy}}$ from the beam line, and the uncertainties $\sigma _\mathrm{R}$ and $\sigma _\mathrm{z}$ provided by the LLP vertex fit. The muon isolation value and the reconstructed mass of the long-lived particles are not used in the MLP classifier; the discrimination power of these two variables is subsequently exploited for the signal determination. The signal training and test samples are obtained from simulated signal events selected under the same conditions as data. A data-driven approach is used to provide the background training samples, based on the hypothesis that the amount of signal in the data is small. For this, a number of candidates equal to the number of candidates of the signal training set, which is of the order of 1000, is randomly chosen in the data. The same procedure provides the background test samples. The MLP training is performed independently for each fully simulated model and dataset. The optimal MLP requirement is subsequently determined by maximizing a figure of merit defined by $\epsilon / \sqrt{N_d + 1}$, where $\epsilon$ is the signal efficiency from simulation for a given selection, and $N_d$ the corresponding number of candidates found in the data.

The generalisation power of the MLP is assessed by verifying that the distributions of the classifier output for the training sample and the test sample agree. The uniformity over the dataset is controlled by the comparison of the MLP responses for several subsets of the data.

The MLP classifier can be biased by the presence of signal in the data events used as background training set. To quantify the potential bias, the MLP training is performed adding a fraction of simulated signal events (up to 5%) to the background set. This test, performed independently for all signal models, demonstrates a negligible variation of the performances quantified by the above figure of merit.

**Fig. 3:**
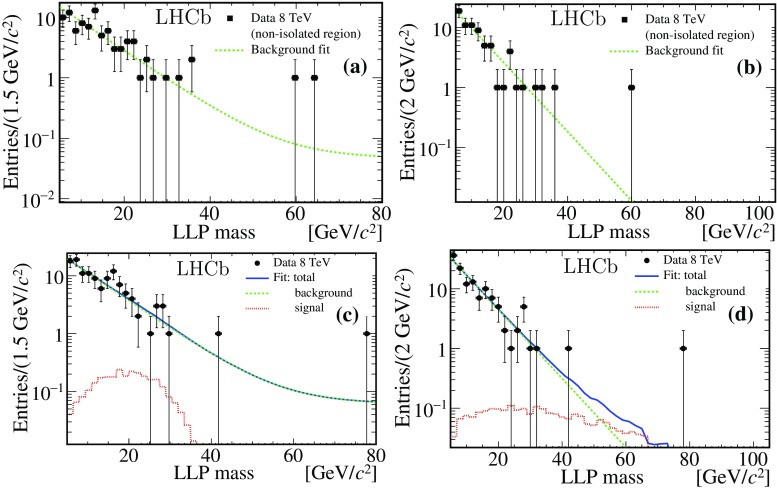
Reconstructed mass of the LLP candidate from the 8 TeV dataset. The *top plots* correspond to events with candidates selected from the background region of the muon isolation variable. They are fitted with the sum of two exponential functions. In the *bottom row* the candidates from the signal region are fitted including a specific signal shape, added to the background component. Subfigures **a** and **c** correspond to the analysis which assumes the LV38 5${\,\mathrm{{ps}}}$ signal model, **b** and **d** are for LV98 10${\,\mathrm{{ps}}}$

## Determination of the signal yield

The signal yield is determined with an extended unbinned maximum likelihood fit to the distribution of the reconstructed LLP mass, with the shape of the signal component taken from the simulated models, plus the background component. After the MLP filter, no simulated background survives; therefore a data-driven method is adopted to determine the background template. The data candidates are separated into a signal region with muon isolation below 1.4 and a background region with isolation value from 1.4 to 2. The signal region contains more than 90% of the signal for all the models considered (see e.g. Fig. 2b). The reconstructed mass obtained from the background candidates is used to constrain an empirical probability density function (PDF) consisting of the sum of two negative slope exponential functions, for which the slope values and amplitudes are free parameters in the fit. The signal PDF is taken from the histogram of the mass distribution obtained from simulation. The fit is performed simultaneously on events from the background region and from the signal region. In the latter the numbers of signal and background events are left free in the fit, while the slope values and the relative strength of the two exponential functions are in common with the background region fit. Examples of fit results are given in Fig. 3, obtained from the $\mathrm 8\,\mathrm{{TeV}}$ dataset for two signal hypotheses, LV38 5${\,\mathrm{{ps}}}$ and LV98 10${\,\mathrm{{ps}}}$. The fitted signal yields, given in Table 1, for both datasets are compatible with the background-only hypothesis.

**Table 1:** Total signal detection efficiency $\epsilon$, including the geometrical acceptance, and numbers of fitted signal and background events, $N_\mathrm{s}$ and $N_\mathrm{b}$, for the different signal hypotheses. The last column gives the value of $\chi ^2/\mathrm {ndf}$ from the fit. The signal models are from the full simulation. Uncertainties are explained in Sect. 6

Dataset	Model	$\epsilon$ %	$N_\mathrm{b}$	$N_\mathrm{s}$	$\chi ^2/\mathrm {ndf}$
$7\,\mathrm{{TeV}}$	LV 38 5${\,\mathrm{{ps}}}$	$0.52 \pm 0.03$	$140.2 \pm 15.5$	$3.8 \pm 10.0$	0.64
	LV 38 10${\,\mathrm{{ps}}}$	$0.57 \pm 0.03$	$115.2 \pm 13.3$	$4.8 \pm 8.2$	1.71
	LV 38 50${\,\mathrm{{ps}}}$	$0.43 \pm 0.02$	$112.9 \pm 13.3$	$9.0 \pm 8.6$	1.50
	LV 98 5${\,\mathrm{{ps}}}$	$0.58 \pm 0.03$	$97.3 \pm 10.3$	$-3.3 \pm 2.4$	0.88
	LV 98 10${\,\mathrm{{ps}}}$	$0.72 \pm 0.04$	$62.6 \pm 8.7$	$-5.6 \pm 2.8$	1.06
	LV 98 50${\,\mathrm{{ps}}}$	$0.56 \pm 0.03$	$99.9 \pm 11.2$	$-3.9 \pm 4.5$	0.33
	LV198 5${\,\mathrm{{ps}}}$	$0.60 \pm 0.04$	$143.8 \pm 12.5$	$-6.9 \pm 2.2$	1.42
	LV198 10${\,\mathrm{{ps}}}$	$0.76 \pm 0.04$	$158.1 \pm 13.1$	$-6.1 \pm 2.7$	1.63
	LV198 50${\,\mathrm{{ps}}}$	$0.66 \pm 0.04$	$118.8 \pm 11.3$	$-0.9 \pm 2.8$	0.89
$\mathrm 8\,\mathrm{{TeV}}$	LV 38 5${\,\mathrm{{ps}}}$	$0.54 \pm 0.04$	$120.3 \pm 15.6$	$2.9 \pm 9.5$	0.74
	LV 38 10${\,\mathrm{{ps}}}$	$0.66 \pm 0.04$	$203.7 \pm 19.9$	$-1.6 \pm 13.3$	0.81
	LV 38 50${\,\mathrm{{ps}}}$	$0.43 \pm 0.02$	$123.3 \pm 15.6$	$3.7 \pm 11.0$	0.99
	LV 98 5${\,\mathrm{{ps}}}$	$0.77 \pm 0.05$	$121.0 \pm 11.2$	$1.0 \pm 2.2$	1.26
	LV 98 10${\,\mathrm{{ps}}}$	$0.96 \pm 0.05$	$123.7 \pm 12.0$	$2.4 \pm 3.4$	0.74
	LV 98 50${\,\mathrm{{ps}}}$	$0.69 \pm 0.04$	$103.8 \pm 10.5$	$2.2 \pm 2.8$	0.94
	LV198 5${\,\mathrm{{ps}}}$	$0.79 \pm 0.06$	$196.3 \pm 14.2$	$-2.3 \pm 2.0$	1.94
	LV198 10${\,\mathrm{{ps}}}$	$1.06 \pm 0.07$	$258.7 \pm 16.2$	$2.3 \pm 2.3$	1.53
	LV198 50${\,\mathrm{{ps}}}$	$0.69 \pm 0.04$	$113.7 \pm 10.8$	$1.3 \pm 2.1$	1.73

The validity of using events with isolation above 1.4 to model the background has been checked by comparing the relevant distributions from events in the background and in the signal regions, including the muon $p_{\mathrm { T}}$ and impact parameter distributions, as well as the number of tracks, invariant mass, vertex $R_\mathrm{{xy}}$ and vertex uncertainties of the LLP candidate. This test is performed with the nominal MLP selection, and also with loosened requirements that result in a threefold increase in the number of background candidates. In both cases all distributions agree within statistical uncertainties, with the $\chi ^2/\mathrm {ndf}$ of the comparison in the range 0.6–1.5.

The sensitivity of the procedure is studied by adding a small number of signal events to the data according to a given signal model. The fitted yields are consistent with the numbers of added events on average, and the pull distributions are close to Gaussian functions with mean values between $-0.1$ and 0.1 and standard deviations on the range from 0.9 to 1.2.

As a final check a two-dimensional sideband subtraction method (“ABCD method” [38]) has been considered. The LLP reconstructed mass and the muon isolation are used to separate the candidates in four regions. The results of this check are also consistent with zero signal for the two datasets.

Both the LLP mass fit and the ABCD methods are tested with *W* and ${\mathrm {Z}}/\gamma$ leptonic decays. Isolated high-$p_{\mathrm { T}}$ muons are produced in such processes with kinematic properties similar to the signal. By removing the minimum $R_\mathrm{{xy}}$ requirement the candidates can be formed by collecting tracks from the primary vertex. As before, the background is taken from a region of muon isolation above 1.4, which contains a negligible amount of signal. For both datasets the number of events obtained from this study is compatible with the cross-sections measured by LHCb [39–41].

## Detection efficiency and systematic uncertainties

The total signal detection efficiency, estimated from fully simulated events, is shown in Table 1. It includes the geometrical acceptance, which for the detection of one $$\tilde{\chi }^{0}_{1}$$ in LHCb is about 11% (12%) at $$\sqrt{s} =7\,\mathrm{{TeV}}$$ (8 TeV). The efficiencies for the models where the fast simulation is used, including processes $$P\!A$$, $$P\!B$$, $$P\!\,C$$, and $$P\!D$$, vary from about 0.1% to about 2%. The efficiency increases with $$m_\mathrm{{LLP}}$$ because more particles are produced in the decay of heavier LLPs. This effect is only partially counteracted by the loss of particles outside the spectrometer acceptance, which is especially likely when the LLP are produced from the decay of heavier states, such as the Higgs-like particles of process $$P\!\,C$$. Another competing phenomenon is that the lower boost of heavier LLPs results in a shorter average flight length, i.e. the requirement of a minimum $$R_\mathrm{{xy}}$$ disfavours heavy LLPs. The cut on $$R_\mathrm{{xy}}$$ is more efficient at selecting LLPs with large lifetimes, but for lifetimes larger than $${\sim }50{\,\mathrm{{ps}}} $$ a considerable portion of the decays falls into the material region and is vetoed. Finally, a drop of sensitivity is expected for LLPs with a lifetime close to the $$b $$ hadron lifetimes, where the contamination from $${b} {\overline{{b}}} $$ events becomes important, especially for low mass LLPs.

A breakdown of the relative systematic uncertainties for the analysis of the $\mathrm 8\,\mathrm{{TeV}}$ dataset is shown in Table 2. The table does not account for the uncertainties associated with the fit procedure, which, as described below, require a specific treatment. The uncertainties on the integrated luminosity are $1.7\%$ for $7\,\mathrm{{TeV}}$ dataset and $1.2\%$ for $\mathrm 8\,\mathrm{{TeV}}$ data [42]. Several sources of systematic uncertainty coming from discrepancies between data and simulation have been considered.

The muon detection efficiency, including trigger, tracking, and muon identification efficiencies, is studied by a tag-and-probe technique applied to muons from $J/\psi \rightarrow {\upmu ^+\upmu ^-}$ [43] and from ${\mathrm {Z}} \rightarrow {\upmu ^+\upmu ^-}$ decays [39–41, 44]. The corresponding systematic effects due to differences between data and simulation are estimated to be between 2.1 and 4.5%, depending on the theoretical model considered.

A comparison of the simulated and observed $p_{\mathrm { T}}$ distributions of muons from ${\mathrm {Z}} \rightarrow {\upmu ^+\upmu ^-}$ decays shows a maximum difference of 3% in the momentum scale; this difference is propagated to the LLP analysis by moving the muon $p_{\mathrm { T}}$ threshold by the same amount. A corresponding systematic uncertainty of 1.5% is estimated for all models under consideration.

The $d_\mathrm{{IP}}$ distribution shows a discrepancy between data and simulation of about 5${\,\upmu \mathrm {m}}$ in the mean value for muons from ${\mathrm {Z}}$ decays, with a maximum deviation of about 20${\,\upmu \mathrm {m}}$ close to the muon $p_{\mathrm { T}}$ threshold. By changing the minimum $d_\mathrm{{IP}}$ requirement by this amount, the change in the detection efficiency is in the range 0.4–1.2%, depending on the model.

**Table 2:** Summary of the contributions to the relative systematic uncertainties, corresponding to the 8 TeV dataset, (the sub-total for the 7 TeV dataset is also given). The indicated ranges cover the fully simulated LV models. The detection efficiency is affected by the parton luminosity model and depends upon the production process, with a maximum uncertainty of 7% for the gluon-gluon fusion process $P\!\,C$. For the fast simulation based analysis there is an additional contribution of 5%. The systematic effects associated with the signal and background models used in the LLP mass fit are not shown in the table

Source	Contribution (%)
Integrated luminosity	1.2
Muon detection	2.1–4.5
Muon $p_{\mathrm { T}}$ scale	1.5
Muon $d_\mathrm{{IP}}$ uncertainty	0.4–1.2
Vertex reconstruction	2.0
Beam line uncertainty	0.2–1.0
MLP training models	1.5–3.6
Muon isolation	2.2
LLP mass scale	0.8–1.5
Models statistics	1.7–2.5
Sub-total $\mathrm 8\,\mathrm{{TeV}}$ dataset	4.9–6.5
(Sub-total $7\,\mathrm{{TeV}}$ dataset	4.9–6.1)
Parton luminosity	3–7
Analysis with fast simulation	5

The vertex reconstruction efficiency is affected by the tracking efficiency and has a complicated spatial structure due to the geometry of the VELO and the material veto. In the material-free region, $$R_\mathrm{{xy}} <4.5\,{\mathrm {mm}} $$, the efficiency to detect secondary vertices as a function of the flight distance has been studied in detail, in particular in the context of the $$b $$ hadrons lifetime measurement [45]. The deviation of the efficiency in simulation with respect to the data is below 1%. For $$R_\mathrm{{xy}}$$ from 4.5$$\,{\mathrm {mm}}$$ to about 12$$\,{\mathrm {mm}}$$ a study performed with inclusive $${b} {\overline{{b}}} $$ events finds differences between data and simulation of less than 5%. The corresponding systematic uncertainties are determined by altering the efficiency in the simulation program as a function of the true vertex position. A maximum of 1% uncertainty is obtained for all the signal models. An alternative procedure to asses this uncertainty considers vertices from $$B^0 \rightarrow {{J/\psi }} {{K} ^{*0}} $$ decays with $${{J/\psi }} \!\rightarrow {\upmu ^+\upmu ^-} $$ and $${{K} ^{*0}} \rightarrow K^+ \pi ^-$$. The detection efficiency in data and simulation is found to agree within 10%. This result, obtained from a four-particle final state, when propagated to LLP decays with on average more than 10 charged final-state particles for all modes, results in a discrepancy of at most 2% between the LLP efficiencies in data and simulation, which is the adopted value for the respective systematic uncertainty.

The uncertainty on the position of the beam line is less than 20${\,\upmu \mathrm {m}}$ [46]. It can affect the secondary vertex selection, mainly via the requirement on $R_\mathrm{{xy}}$. By altering the PV position in simulated signal events, the maximum effect on the LLP selection efficiency is in the range 0.2–1%.

The imprecision of the models used for training the MLP propagates into a systematic difference of the detection efficiency between data and simulation. The bias on each input variable is determined by comparing simulated and experimental distributions for muons and LLP candidates from $\mathrm {Z}$ and $\mathrm {W}$ events, and from ${b} {\overline{{b}}}$ events. The effect of the biases is subsequently estimated by testing the trained classifier on altered simulated signal events: each input variable is modified by a scale factor randomly drawn from a Gaussian distribution of width equal to the corresponding bias. The RMS variation of the signal efficiency distributions after the MLP range from 1.5 to 3.6% depending on the signal model. These values are taken as contributions to the systematic uncertainties.

The signal region is selected by the requirement of a muon isolation value lower than 1.4. By a comparison of data and simulated muons from $\mathrm {Z}$ decays, the uncertainty on this variable is estimated to be $\pm 0.05$. This uncertainty is propagated to a maximum 2.2% effect on the detection efficiency.

Comparing the mass distributions of ${b} {\overline{{b}}}$ events selected with relaxed cuts, a maximum mass scale discrepancy between data and simulated events of 10% is estimated. The corresponding shift of the simulated signal mass distribution results in a variation of the detection efficiency between 0.8 and 1.5%.

The statistical precision of the efficiency value determined from the simulated events is in the range 1.7–2.5% for the different models.

The theoretical uncertainties are dominated by the uncertainty of the partonic luminosity. Their contribution to the detection efficiency uncertainty is estimated following the procedure explained in Ref. [44] and vary from 3% up to a maximum of 7%, which is found for the gluon-gluon fusion process $P\!\,C$.

For the analysis based on the fast simulation, a 5% uncertainty is added to account for the difference between the fast and the full simulation, as explained in Sect. 3.

The choice of the background and signal templates can affect the results of the LLP mass fit. The uncertainty due to the signal model accounts for the mass scale, the mass resolution and the finite number of events available to construct the model. Pseudoexperiments in which 10 signal events are added to the data are analysed with a modified signal template, and the resulting number of fitted candidates is compared to the result from the nominal fit model. Assuming as before a 10% uncertainty on the signal mass scale, a maximum absolute variation of 0.6 fitted signal candidates is obtained. No significant effects are obtained by modifying the signal mass resolution with an additional smearing. Changing the statistical precision by reducing the initial number *N* of signal events used to build the histogram PDF by $2 \sqrt{N}$ has no significant effect either.

The uncertainty induced by the choice of the background model is obtained by reweighting the candidates from the background region in such a way that the distribution of the number of tracks included in the LLP vertex fit exactly matches the distribution in the signal region. This test is motivated by the fact that the number of tracks has a significant correlation with the measured mass. The fits of the mass distribution of pseudoexperiments give absolute variations in the numbers of fitted signal events in the range 0.1–1.6, the largest value at low LLP mass. Reweighting the candidates in such a way as to match the $p_{\mathrm { T}}$ distributions gives variations which are less than 0.5 events for all models. Moving the isolation threshold by $\pm 0.1$ leads to variations of the order of 0.01 events. In conclusion, the variation on the number of fitted candidates associated to the choice of the PDF models is in the range of 1–2 events. The calculation of the cross-section upper limits takes into account this uncertainty as an additional nuisance parameter on the fit procedure.

## Results

**Fig. 4:**
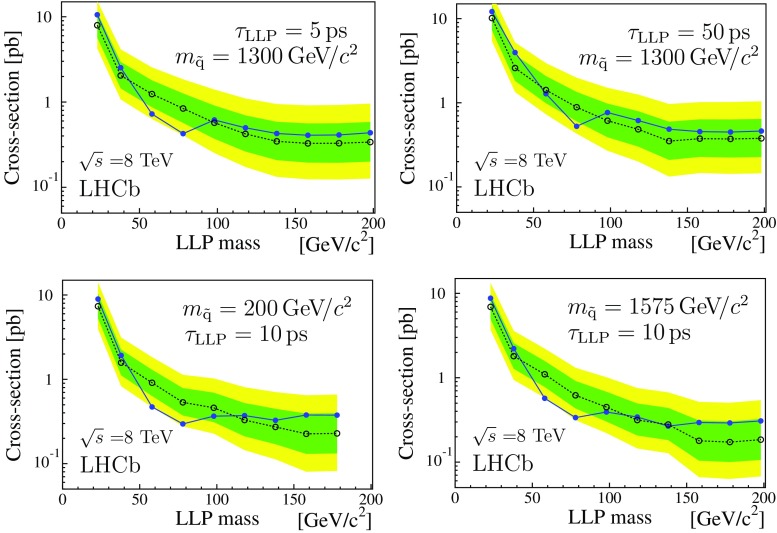
Expected (*open dots* with 1$\sigma$ and 2$\sigma$ bands) and observed (*full dots*) cross-section times branching fraction upper limits at 95% confidence level, as a function of the LLP mass from the 8 TeV dataset. The theoretical models assume the full set of SUSY production processes available in Pythia  6 with default parameter settings, unless otherwise specified. The gluino mass is 2000 $\mathrm{{GeV}}/c^2$

**Fig. 5:**
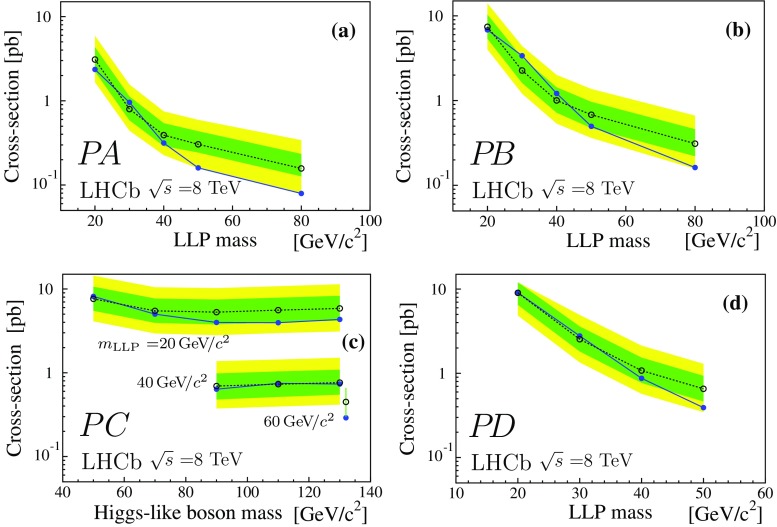
Expected (*open dots* and 1$\sigma$ and 2$\sigma$ bands) and observed (*full dots*) cross-section times branching fraction upper limits (95% CL) for the processes indicated in the bottom left corner of each plot, $\tau _\mathrm{{LLP}}$ is always 10${\,\mathrm{{ps}}}$. The results correspond to the 8 TeV dataset. **a** Upper limits as a function of the LLP mass for process $P\!A$; **b** as a function of the LLP mass for process $P\!B$, with $m_{\text {``}\tilde{\mathrm{g}}\text {''}} =100$ $\mathrm{{GeV}}/c^2$; **c** as a function of $m_\mathrm{h^0}$ for process $P\!\,C$ for $m_\mathrm{{LLP}}$ of 20, 40, and 60 $\mathrm{{GeV}}/c^2$, from *top* to *bottom* (the single point at 130 $\mathrm{{GeV}}/c^2$ with $m_\mathrm{{LLP}} =60\,\mathrm{{GeV}}/c^2$ has been shifted to the right for visualisation); **d** upper limits as a function of the LLP mass for process $P\!D$ with $m_{\tilde{\mathrm{q}}} =60$ $\mathrm{{GeV}}/c^2$

**Fig. 6:**
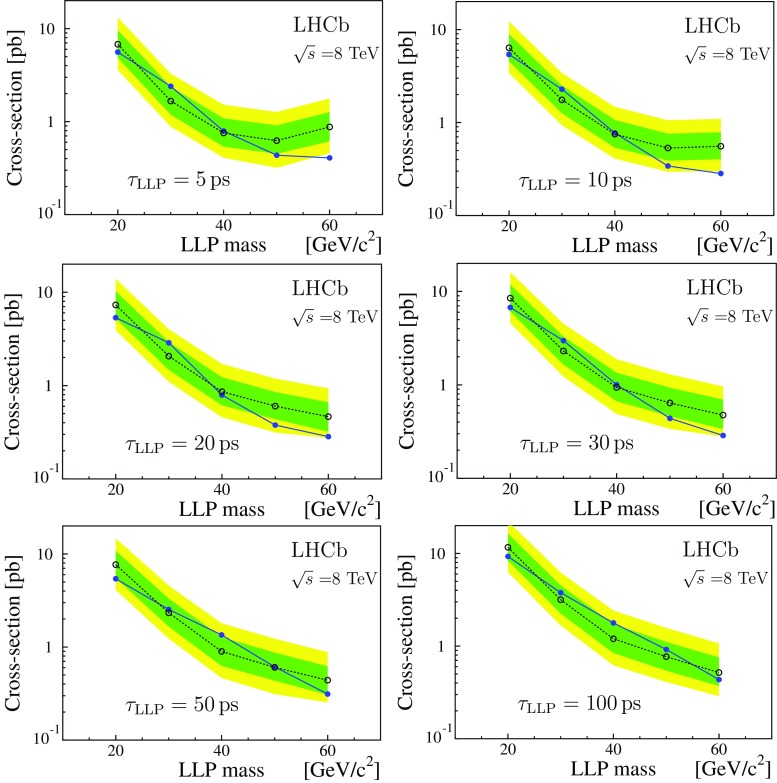
Expected (*open dots* with 1$\sigma$ and 2$\sigma$ bands) and observed (*full dots*) cross-section times branching fraction upper limits (95% CL) for the processes $P\!\,C$ as a function of the LLP mass; the LLP lifetime $\tau _\mathrm{{LLP}}$ is indicated in each plot, $m_\mathrm{h^0} =125\,\mathrm{{GeV}}/c^2.$ The results correspond to the 8 TeV dataset

The LLP candidates collected at $\sqrt{s} =7$ and 8 TeVare analysed independently. The fast simulation is used to extend the MSSM/mSUGRA theoretical parameter space of the LV models, and for the analysis of processes $P\!A$, $P\!B$, $P\!\,C$, and $P\!D$. The results obtained are found to be compatible with the absence of signal for all signal model hypotheses considered. The 95% confidence level (CL) upper limit on the production cross-sections times branching fraction is computed for each model using the CLs approach [46]. The numerical results for the fully simulated LV models are given in Table 3.^1^ A graphical representation of selected results is given in Figs. 4, 5 and 6.

The MSSM/mSUGRA LV models are explored by changing the common squark mass and the gluino mass. Figure 4 gives examples of the cross-section times branching fraction upper limits as a function of $m_\mathrm{{LLP}}$ for such models for two values of $\tau _\mathrm{{LLP}}$, and two values of the squark mass. The gluino mass is set to 2000 $\mathrm{{GeV}}/c^2$. Varying the gluino mass from 1500 to 2500 $\mathrm{{GeV}}/c^2$ has almost no effect on the results. The decrease of sensitivity for decreasing $m_\mathrm{{LLP}}$ is explained by the above-mentioned effects on the detection efficiency.

A representation of selected results from the processes $P\!A$, $P\!B$, $P\!\,C$, and $P\!D$ is given in Fig. 5. The single LLP production of $P\!B$ has a lower detection probability compared to the double LLP production case, $P\!A$, which explains the reduced sensitivity. The $P\!B$ plots correspond to $m_{\text {``}\tilde{\mathrm{g}}\text {''}} =100\,\mathrm{{GeV}}/c^2$. Varying $m_{\text {``}\tilde{\mathrm{g}}\text {''}}$ from 100 to 1000 $\mathrm{{GeV}}/c^2$ decreases the detection efficiency by a factor of two, while an increase by a factor of two is obtained reducing $m_{\text {``}\tilde{\mathrm{g}}\text {''}}$ to 20 $\mathrm{{GeV}}/c^2$. The results for process $P\!\,C$ are given as a function of the Higgs-like boson mass, for three values of $m_\mathrm{{LLP}}$. Again the sensitivity of the analysis drops with decreasing $m_\mathrm{{LLP}}$. The results shown for $P\!D$ are for $m_{\tilde{\mathrm{q}}} =60\,\mathrm{{GeV}}/c^2$, which limits the maximum $m_\mathrm{{LLP}}$ value. In process $P\!D$ some of scattering energy is absorbed by an additional jet during the LLP production, reducing the detection efficiency by a factor of two with respect to $P\!A$. Finally, Fig. 6 gives the cross-section upper limits times branching fraction as a function of $m_\mathrm{{LLP}}$, for the process $P\!\,C$ with a mass of 125 $\mathrm{{GeV}}/c^2$ for the Higgs-like boson and LLP lifetime from 5 to 100${\,\mathrm{{ps}}}$. These results can be compared to the prediction of the Standard Model Higgs production cross-section of about 21$\,{\mathrm {pb}}$ at $\sqrt{s} =8\,\mathrm{{TeV}}$ [46].

**Table 3:** Upper limits (95% CL) on the production cross-section times branching fraction (pb) for the $7\,\mathrm{{TeV}}$ and $\mathrm 8\,\mathrm{{TeV}}$ datasets, based on the fully simulated LV signal samples

	$7\,\mathrm{{TeV}}$ dataset		$\mathrm 8\,\mathrm{{TeV}}$ dataset	
Model	Expected	Observed	Expected	Observed
LV 38 5${\,\mathrm{{ps}}}$	4.03$^{+1.79}_{-1.20}$	4.73	2.04$^{+0.89}_{-0.60}$	2.32
LV 38 10${\,\mathrm{{ps}}}$	2.95$^{+1.36}_{-0.89}$	3.76	2.24$^{+0.95}_{-0.65}$	2.13
LV 38 50${\,\mathrm{{ps}}}$	4.08$^{+1.89}_{-1.24}$	6.15	2.86$^{+1.23}_{-0.83}$	3.10
LV 98 5${\,\mathrm{{ps}}}$	1.78$^{+0.97}_{-0.60}$	1.21	0.62$^{+0.36}_{-0.22}$	0.57
LV 98 10${\,\mathrm{{ps}}}$	1.52$^{+0.78}_{-0.49}$	0.94	0.52$^{+0.27}_{-0.17}$	0.53
LV 98 50${\,\mathrm{{ps}}}$	2.21$^{+1.10}_{-0.70}$	1.83	0.70$^{+0.41}_{-0.25}$	0.77
LV198 5${\,\mathrm{{ps}}}$	1.50$^{+0.86}_{-0.52}$	0.95	0.59$^{+0.34}_{-0.21}$	0.40
LV198 10${\,\mathrm{{ps}}}$	1.18$^{+0.68}_{-0.41}$	0.85	0.27$^{+0.20}_{-0.11}$	0.42
LV198 50${\,\mathrm{{ps}}}$	0.92$^{+0.67}_{-0.38}$	1.07	0.52$^{+0.35}_{-0.21}$	0.58

## Conclusion

Long-lived massive particles decaying into a muon and two quarks have been searched for using proton-proton collision data collected by LHCb at $\sqrt{s} =7$ and 8 TeV, corresponding to integrated luminosities of 1 and 2 $\,\hbox {fb}^{-1}$, respectively. The background is dominated by ${b} {\overline{{b}}}$ events and is reduced by tight selection requirements, including a dedicated multivariate classifier. The number of candidates is determined by a fit to the LLP reconstructed mass with a signal shape inferred from the theoretical models.

LHCb can study the forward region $2<\eta <5$, and its low trigger $p_{\mathrm { T}}$ threshold allows the experiment to explore relatively small LLP masses. The analysis has been performed assuming four LLP production mechanisms with the topologies shown in Fig. 1, covering LLP lifetimes from 5 ps up to 100${\,\mathrm{{ps}}}$ and masses in the range 20–80  $\mathrm{{GeV}}/c^2$. One of the processes proceeds via the decay of a Higgs-like particle into two LLPs: the mass of the Higgs-like particle is varied between 50 and 130 $\mathrm{{GeV}}/c^2$, comprising the mass of the scalar boson discovered by the ATLAS and CMS experiments. In addition, the full set of neutralino production mechanisms available in Pythia in the context of MSSM/mSUGRA has been considered, with an LLP mass range 23–198  $\mathrm{{GeV}}/c^2$. The results for all theoretical models considered are compatible with the background-only hypothesis. Upper limits at 95% CL are set on the cross-section times branching fractions.

## Appendix

### Parameters of the fully simulated signal models

The parameters used to generate nine fully simulated signal samples in the context of MSSM/mSUGRA are given in Table 4. Other MSSM parameters remain at their default Pythia values. The lightest neutralino, $\tilde{\chi }^{0}_{1}$, decays via the lepton number violating mode LQD (for the definition see [3, 14]). As an approximation, equal branching fractions are assumed for all QD pairs, except for the pairs with a top quark, which are excluded.

**Table 4:** Parameters for the generation of the nine fully simulated signal models. The LLP is the lightest neutralino, $\tilde{\chi }^{0}_{1}$ with $m_{\tilde{\chi }^{0}_{1}} = m_\mathrm{{LLP}}$; $M_1$ and $M_2$ are the Pythia parameters RMSS(1) and RMSS(2), $m_{\tilde{\mathrm{g}}}$ is RMSS(3), $\mu$ is RMSS(4), $\tan {\beta }$  RMSS(5) and $m_{\tilde{\mathrm{q}}}$ is RMSS(8-12). Samples with lifetime of 5, 10 and 50${\,\mathrm{{ps}}}$ have been produced for each mass

Model	$M_1$ $[\,\mathrm{{GeV}}/c^2 ]$	$M_2$ $[\,\mathrm{{GeV}}/c^2 ]$	$m_{\tilde{\mathrm{g}}}$ [ $\mathrm{{GeV}}/c^2$ ]	$\tan {\beta }$	$\mu$	$m_{\tilde{\mathrm{q}}}$ [ $\mathrm{{GeV}}/c^2$ ]	$m_{\tilde{\chi }^{0}_{1}}$ [ $\mathrm{{GeV}}/c^2$ ]
LV38 5/10/50ps	40	2000	2000	2.0	1200	1300	38
LV98 5/10/50ps	100	2000	2000	2.0	1200	1300	98
LV198 5/10/50ps	200	2000	2000	2.0	1200	1300	198

Two sets of events have been produced with $\sqrt{s}$ =7 and 8 TeV. Only events with one muon and one $\tilde{\chi }^{0}_{1}$ in the LHCb acceptance are processed in Geant4, corresponding to about 11% of the $\sqrt{s}$ =7 TeV generated events, 12% at 8 TeV.

## Footnotes

1 The numerical results for all the other models are available as supplementary material.
